# Forward to Bernstein: Movement Complexity as a New Frontier

**DOI:** 10.3389/fnins.2020.00553

**Published:** 2020-06-03

**Authors:** Elena Biryukova, Irina Sirotkina

**Affiliations:** ^1^Laboratory of Mathematical Neurobiology of Learning of Institute of Higher Nervous Activity and Neurophysiology, Russian Academy of Sciences, Moscow, Russia; ^2^Research Institute of Translational Medicine, N.I. Pirogov Russian National Research Medical University, Moscow, Russia; ^3^Center for the History of Organization of Science and of Science Studies, S.I. Vavilov Institute for the History of Science and Technology, Russian Academy of Sciences, Moscow, Russia

**Keywords:** N. A. Bernstein, complex movement, degree of freedom, motor control, coordination

## Abstract

The paper attempts to demonstrate that the “old-school” approach in motor control studies suggested over a century ago by I. M. Sechenov (1866/1968, 1901) and, later, N. A. Bernstein (1923, 1929, 1940, 1961) remains valid and relevant. Their methodology was to study the motor “periphery” in order to determine “central” mechanisms of motor control. The approach, which can be termed “bottom-up,” is contrasted with the “top-down” methodology of first making models of brain control and then investigating the functioning of muscles and joint torques. The earlier progress in motor control studies was, to a great extent, due to the fact that Bernstein developed procedures to register multiple degrees of freedom and thus to analyze in detail the structure of natural movement. The analysis of multi-joint goal-directed movement *per se*, in its own right, could be the starting point for productive studies of both muscular system functioning and its central control by the nervous system. The article reports on how, in some of his less well known works, Bernstein analyzed complex multi-joint movements. The article’s main focus is on movements of the arm as a model example of multi-joint goal-directed movements. It reviews a body of research that follows the “bottom-up” tradition by summarizing contemporary research on two contrasting cases: (1) of a highly coordinated motor skill, as achieved in musical performance or in a precise stroke; and (2) of pathological arm movement in post-stroke neurological patients who have lost capacity as a result of damage to the central nervous system. The paper demonstrates the need for inclusive analyses of all existing degrees of freedom of the moving arm. In the first case, this is important in order to identify some features of learning skills. In the second case, it is important in order to adequately assess the restoration of movements in the process of rehabilitation. The paper concludes by arguing that the “bottom-up” approach in studying the nervous control of complex movements possess a heuristic potential that has not been exhausted.

## Introduction

As Herbert Spencer claimed already in the mid-nineteenth century, in evolution organisms became more and more complex. In his famous essay, “Progress: Its Law and Cause,” Spencer referred to “the same evolution of the simple into the complex, through successive differentiations” and claimed: “From the earliest traceable cosmic changes down to the latest results of civilization, we shall find that the transformation of the homogenous into the heterogeneous, is that in which progress essentially consists” (Spencer, 1868, p. 3). The more complex the organism, the longer it takes for it to mature and the longer the period of upbringing. In the course of evolution, individual organisms increase their ability to learn from experience and to store information in memory. Complexity and the learning ability go hand-in-hand: organisms have more and more “value,” as more and more “biological capital” is invested in them (Gould, 1977). Yet, in the course of a longer period of upbringing and education, they also become more vulnerable.

The physicists Prigogine and Stengers (1984) criticized the natural sciences for reducing complexity instead of recognizing it and making it the point of departure in research. By contrast, their own theory of *thermodynamic systems far from equilibrium* included complexity and developed the notion of “metastability” (in 1977 Prigogine received the Nobel Prize for his work on irreversible thermodynamics of unstable systems). At a certain evolutionary stage, *metastability* signifies the capacity of organisms for learning. The capacity to learn distinguishes, for instance, populations of flies living and dying by the million without any apparent signs of learning, and a population of primates every member of which combines individual experience with collective experience of the entire population.

The same general point that Prigogine and Stengers made, the Russian physiologist Nikolai Aleksandrovich Bernstein (1896–1966) argued in detail in relation to motor control in living beings. He showed how movements evolve in terms of increased adaptability, flexibility of skills, and trainability. In particular, he formulated the hypothesis of various *levels of motor control*. He claimed that in the course of evolution new, more complex movements appear and new levels of motor control are added. The more recent in evolution the level, the higher the significance and complexity of tasks accessible at this level, and the more flexible, adaptable, and trainable the organism’s movements (Bernstein, 1947). The evolution therefore unfolds from a complete absence of learning capacity – with movements limited to a few inborn forms of coordination, like in insects – to the ability to give one-off, “impromptu” responses to unpredictable and unusual tasks. The larger scope of available movements increases the organism’s chances in the struggle for survival. As a person with an excellent sense of humor and a talent for popular explanation, Bernstein illustrated the absence of learning skills in insects by the case of a trained, performing flea. He mentioned a circus trick where fleas pulled a tiny carriage with a canon attached to it (at some stage, the canon even fired). The trainer revealed the secret: fleas were kept in a flat box with a glass cover where they could not jump. When their back leg muscles weakened from lack of use, and the fleas could only crawl, the trainer easily harnessed them to a carriage. It was in fact a trick, and not training, and Bernstein argued that the latter is hardly possible in insects (Bernstein, 1947/1991, p. 206).

Bernstein’s experimental research was tightly connected to the new ways of recording motion that appeared at the late nineteenth century, such as the technique of *chronophotography*, a sequence of photo shots taken with equal time intervals (Marey, 1868, 1873). Bernstein preferred to use *in vivo* techniques rather than *in vitro* experiments. He recorded work operations like hammering, everyday movements (eating with a spoon), music playing (varying tempo and sound intensity), and also various kinds of locomotion, including the walking of neurological patients, old people, babies, and older children, comparing their movements to locomotion in healthy adults. The method of *natural experiment* helped to find the optimum solution in performing various work tasks and to examine the structure of normal and pathological walking. In order to analyze movement, Bernstein calculated velocities, accelerations, and muscle torques in each joint of the moving body on the basis of spatial coordinates of joints. He termed the method “*cyclometry*” (measuring cycles of movement). Operationally, it meant drawing charts for every recorded joint movement on the time axis. The method of calculating movement parameters was labor-intensive and could require months of work. Yet at the end the kinematic picture of the movement became clear, and it was possible to discern dynamic impulses coming from the central nervous system acting on the periphery of movement (for instance, as a result of the contact of the hand with the object as in a working stroke, or of the feet with the ground in locomotion). Over three to four decades, Bernstein and his team recorded and analyzed hundreds of movements, including hand operations, healthy and pathological walking and running, athletic movements, and many other kinds of human and animal motor activity.

Bernstein therefore made the camera and calculator, as opposite to the lancet, powerful instruments for a thorough examination of nervous control in intact natural movements. In this, he followed the Russian physiologist Ivan Mikhailovich Sechenov who, in the early 1860s, formulated the program of studying the nervous system on the basis of analyzing the movement, the final outcome of motor control. In his fundamental work, *Reflexes of the Brain* (Sechenov, 1866/1968, p. 265), he wrote: “…it becomes clear to the reader, once and for all, that absolutely all such qualities of the external manifestation of brain activity which we characterize as animation, passion, mockery, grief, joy, etc., are nothing more than the results of a greater or lesser contraction of some group of muscles – a purely mechanical act, as everyone knows. Even the most confirmed spiritualist must concede this. Indeed, how could it be otherwise, when we know that from a soulless instrument the hand of the musician tears out sounds full of passion and life, and that the hand of the sculptor brings life into stone. Both the hand of the musician and that of the sculptor, creating life, is capable only of mechanical movement which, strictly speaking, can even be subjected to mathematical analysis and expressed by a formula… If the reader considers this, he will agree that the time must come when people will be able to analyze the external manifestations of the activity of the brain just as easily as the physicist now analyses a musical chord…”

About the same time as Sechenov, the French scientist Etienne-Jules Marey, one of the authors of *chronophotography*, invested big hopes in deciphering brain mechanisms with the help movement analysis: “… I believe that *movement is the most important act to the execution of which all other functions contribute*. Moreover, today, we should consider movement in a wider sense and relate to it a large number of changing states, which equally could be studied by the graphical method. The essence of the movement – muscle contraction and the function of the central nervous system – was till now the most mysterious field of biology; it will soon be the most studied” (pp. VI–VII) (translation and italics are ours – *E.B., I.S*.) (Marey, 1868).

One of the most important of Sechenov’s steps toward the program of analyzing brain through movement was the formulation of the “bottom-up” approach to motor control in his later work, *Essay on Human Working Movements* (Sechenov, 1901). The human motor machine consists of three parts: bone structure, or the skeleton, the constellation of muscles connected to the bones, and the nervous system controlling muscles. Sechenov calculated static muscular efforts, subdividing them into functional components which ensure efficient and stable arm positions during performance of working movements. His analysis was based on the laws of mechanics as well as on anatomical data of the arm joints structure, including areas in which the muscles are attached to the bones.

Bernstein developed Marey’s chronophotography further and, after having explored a similar class of working movements, made the next step toward the analysis of the nervous control of movements. Research into complex multi-joint movements that Bernstein carried out in the 1920s and 1930s stimulated him to review in detail the contemporary state of the art in movement physiology. He wrote this up in a manuscript that was completed in 1937 but for a long time remained unpublished (Bernstein, 1937/2003). Soon thereafter he also formulated his own theory of what happens when the nervous system controls various movements (Bernstein, 1947).

It is important that Bernstein started his pioneering research from complex movements of the arm, including work operations of the hand, with various instruments (Bernstein, 1923). The examination of *natural* human movements clearly requires the analysis of complex coordination and control of multiple *degrees of freedom* (DoFs).

In the review below, we attempt to trace the presence of the bottom-up approach in contemporary studies of two contrasting cases, (1) of a highly coordinated motor skill, and (2) of pathological arm movement in post-stroke neurological patients who have lost capacity as a result of damage to the central nervous system. For both cases, the conclusion about future research is the same: forward to Bernstein.

## Bernstein’s Research: From Kinematic Analysis to Muscular Work and Nervous Control

### Hammer Stroke

How are DoFs of the arm organized in a coordinated goal-directed movement? As early as 1923, Bernstein suggested a means in his paper, “Studies of biomechanics of the strike with the camera recording” (Bernstein, 1923). The Latvian specialist in biomechanics and, until recently, the keeper of Bernstein’s archive, Harald Janson, writes: “It appears almost improbable that a young doctor, who had just touched science for the first time, could write a groundbreaking work that laid out the path to all his future research. The central part of the article is analytic in character and written at a professional level of mathematics and physics. It provides formal analysis of human movements. In a single energetic stroke, the technique of processing photographic images of movement had been put at an advanced academic stage” (Janson, 1992). We know, however, that already in a Gymnasium (a German-type high school), the young Bernstein was deeply interested by mathematics, and as a student of the Moscow University Medical School, he followed courses in mathematics. Later, he estimated his knowledge of the subject “at the level of a rank-and-file mathematics professor” (Sirotkina, 2018).

**FIGURE 1:**
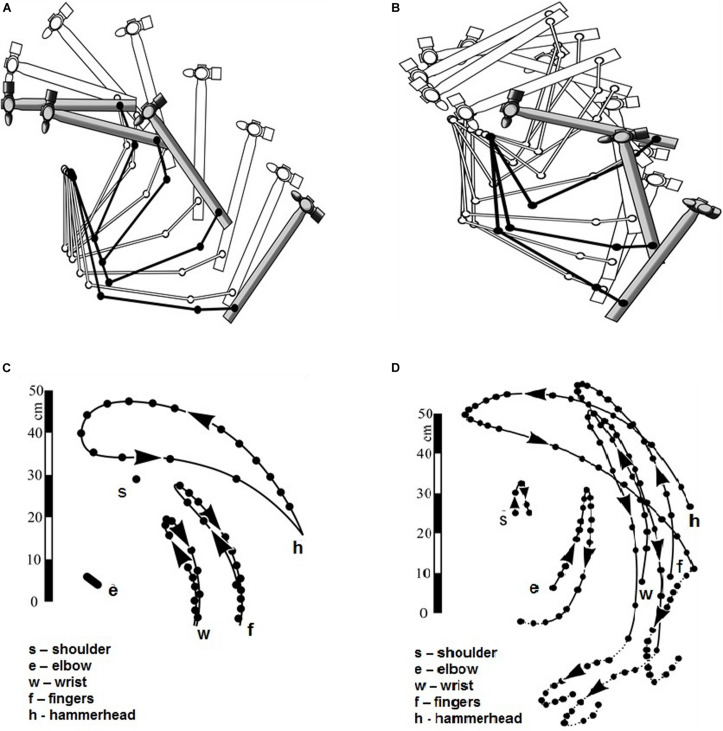
**(A,B)** Positions of the arm links and the hammer during abductive stroke **(A)** and vertical stroke **(B)**. Hammer positions during the lifting are shown in white and during the descent in gray. **(C,D)** Cyclogamms of the abductive stroke **(C)** and of the vertical stroke **(D)**. (Reproduced from Bernstein, 1923. Public domain).

**FIGURE 2:**
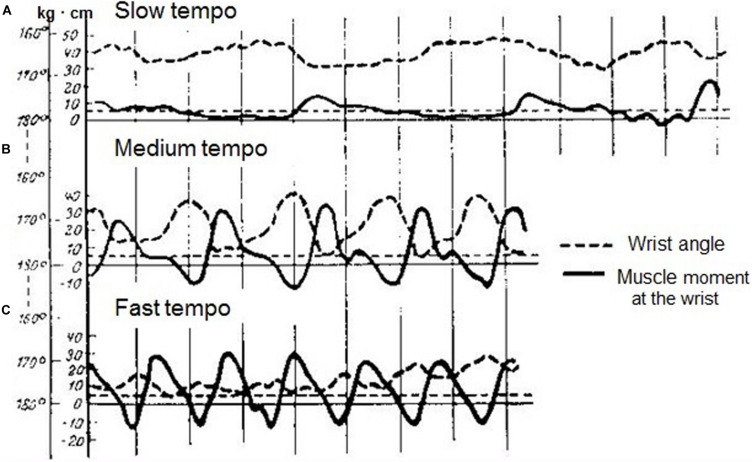
Time courses of wrist angle (dotted lines) and wrist torques (solid lines) during octave execution at a slow **(A)**, a medium **(B)** and a fast **(C)** tempo. (Reproduced from Bernstein and Popova, 1930. Public domain).

When analyzing the hammer stroke (Bernstein, 1923), two different forms of the stroke were compared: (1) a stroke involving the abduction in the shoulder (Figures 1A,C), and (2) a vertical stroke when all the arm’s links remain within the vertical plane (Figures 1B,D). The criterion for comparison is the final velocity of the hammerhead which determines the power of the stroke. The most efficient stroke should minimize those joints movements which do not contribute to the velocity of the hammerhead. The registration of the stroke with the abduction (Figures 1A,C) and the vertical stroke (Figures 1B,D) has shown that:

1) In the stroke with shoulder abduction the amplitudes of the joints movements are smaller and the amplitude of the hammerhead is larger than in vertical stroke, which means that the stroke with abduction is more efficient.
2) In the stroke with shoulder abduction the center of gravity of the hammerhead lifts higher, and the centers of gravity of the arm chains are located lower than in vertical stroke. Given that potential energy of the hammerhead during the stroke is transformed into kinetic energy, and potential energy of the arm chains is not, the stroke with shoulder abduction appears more efficient.
3) The velocities of the arm chains and the hammerhead can be easily read on cyclogramms (Figures 1C,D): they are proportional to the distance between the nearest points registered. For the stroke with shoulder abduction the velocity of the hammerhead is higher than velocities of the joints (Figure 1C). For vertical stroke, they are comparable, which once again speaks in favor of the stroke with abduction in the shoulder (Figure 1D).
4) Finally, in vertical stroke there occurs a recoil, or kickback, in which part of the hammerhead energy is wasted. In the stroke with shoulder abduction this is not the case.

In an exact accordance with Sechenov’s method (Sechenov, 1901), Bernstein used anatomical data on directions of muscle forces in order to analyze their functioning in the performance of two types of stroke. Bernstein explained the superior efficiency of the shoulder stroke over the vertical one by the fact that during the shoulder abduction the direction of the net force for the strongest shoulder muscle, the deltoid (m. deltoideus), coincides with the direction of the entire arm movement. By contrast, in vertical stroke the direction of the arm coincides with the net force of the weakest muscle, m.coracobrachialis, and the force of the deltoid is only partially used.

### The Piano Strike

In the late 1920s Bernstein worked in a unique institution (unfortunately a rather short-lived one), the State Institute for the Music Sciences (SIMS). His research in the institute dealt with the character of the piano strike and its training. Being himself a fine pianist, Bernstein believed that the strike of piano keys depended chiefly on movements in the elbow and wrist joints rather than the fingers’ movements. He registered all these movements with the help of chronophotography. On this basis, he calculated joint torques and their coordination (Bernstein and Popova, 1930; translated in Kay et al., 2003).

He was able to invite as experimental subjects two outstanding pianists, the Russian, Konstantin Igumnov, and the Dutchman, Egon Petri. In order to exclude the emotional aspect, the subjects were asked to perform simple rhythmical movements on the keyboard. To study the power of the piano strike, they had to perform monotonous octaves while altering the force of the strike, from pianissimo to fortissimo. To study the temporal structure of the piano strike, they were asked to take octaves in different tempos, from slower to faster, and faster ending by prestissimo.

It had been demonstrated that the coordination between the elbow and the wrist joint torques did not depend on the force of the strike, yet it cardinally depended on the tempo of the performance (Figure 2). With a slow tempo, the moments of force in the joints alter independently from each other, and they are separated by the periods of “silence” (Figure 2A). With increased tempo, they come to coordinate with each other (Figure 2B). Under a higher tempo (over 400 key strikes per minute), they are well approximated by an elastic oscillator performing the passive movement due to segments inertia and wrist joint rigidity (Figure 2C). Bernstein stress that oscillatory movement of the hand does not represent a free oscillation, but a forced oscillation induced by the forearm and the upper arm muscle forces.

As usual in his studies, Bernstein concluded with practical recommendations. Given that the synergy of moments of forces in the joints significantly alters under higher tempos, it is wrong to begin learning fast passages by playing them slowly. The two skills have a different structure, and the nervous control is also different. Bernstein rejected the common belief that the pianist minimizes muscular effort by using passive arm movement under the force of gravity. He demonstrated that in fast tempo such a passive movement is not possible, for reasons which are purely mechanical, and in slow tempo the falling of the arm happens very rarely, though theoretically it can occur.

Bernstein’s study of the piano strike certainly appeared a pioneering work, yet he was far from being the first to register and apply mathematical analysis to music playing. Thus, Marey’s assistant and an amateur violinist, Georges Demenÿ, chronophotographed his own performance on the violin (amongst many other kinds of human and animal movements). In his work, “Le violoniste” (Demenÿ, 1905), Demenÿ described what appeared at that time to be exceptional results: coordination of several joints allows the performance of a straight movement of the violin bow, and the method to assess the skill of playing the violin is by studying the character of performing staccato. Demenÿ’s study, as well as the work by Bernstein, preceded contemporary biomechanics of music performance by almost a century.

### Eating With a Spoon

**FIGURE 3:**
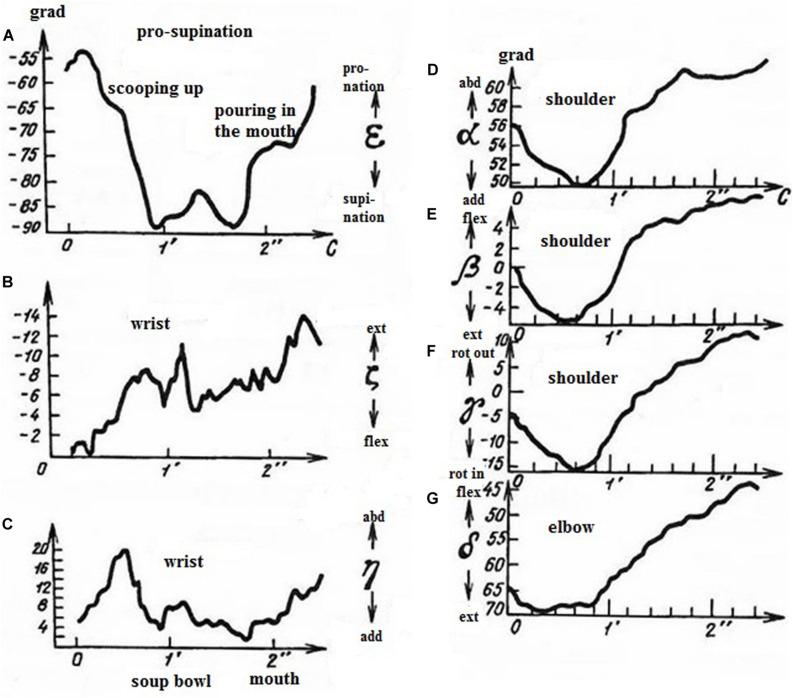
Time courses of arm joint angles during eating with a spoon – scooping up and pouring in the mouth. Axis Y – joint angles, degrees. Axis X – time in seconds (1 – soup bowl; 2 – mouth). **(A)** forearm pronation (scooping up) – supination (pouring in the mouth), **(B)** extension in the wrist (during the whole period of eating); **(C)** wrist abduction (scooping up) – adduction (pouring in the mouth); **(D)** shoulder adduction (scooping up) – abduction (pouring in the mouth); **(E)** shoulder flexion (scooping up) – extension (pouring in the mouth); **(F)** shoulder internal (scooping up) – external (pouring in the mouth) rotation; **(G)** elbow extension (scooping up) – flexion (pouring in the mouth). (Reproduced from Bernstein and Salzgeber, 1948. Public domain).

The same methodological principle was applied to the analysis of arm movement during eating with a spoon (Bernstein and Salzgeber, 1948). Analysis of seven arm DoFs made it possible to reveal three independent kinematic synergies testifying to a well-learned coordination: pronation-supination of the forearm (Figure 3A), extension-flexion and abduction-adduction in the wrist (Figures 3B,C), and synergy of three rotations in the shoulder and extension-flexion in the elbow (Figures 3D–G). Bernstein and Salzgeber examined mechanical work produced by the muscles involved in each synergy and made suggestions for the construction of a prosthesis of the arm minimizing muscle energy expenditure (Bernstein and Salzgeber, 1948).

It is important that movements chosen for examination were natural and responded to a significant functional goal. The description of optimal muscle functioning in Bernstein (1923) became possible due to taking into analysis an additional DoF, shoulder abduction. Taking into account of all the arm DoFs allowed description of three independent synergies assuring a natural movement (Bernstein and Salzgeber, 1948). The analysis of the kinematic and dynamic structure of the piano strike clarified the form of nervous control crucially related to movement velocity (Bernstein and Popova, 1930).

## Post-Bernsteinian “Bottom-Up” Studies of Complex Natural Movements

### Movement Organization in Music Performance

In the period after Bernstein “bottom-up” studies of functional movements in natural conditions to some extend gave way to “top-down” studies. The latter start from making models of brain control and afterward proceed to investigating muscles functioning and joint torques (see Bizzi et al., 1991; Feldman and Levin, 1995; Kawato, 1999; Todorov and Jordan, 2002 as the examples of most important “top-down” studies). The most frequent movement tests used in “top-down” frame concern typing, reaching and grasping performed in a special laboratory setting (Rosenbaum, 2009). Recently, however, there is an increased interest in complex natural movements as a source for understanding motor organization. Above all, this concerns studies of the movements of the arm playing musical instruments (Winold et al., 1994; Turner-Stoker and Reid, 1999; Rasamimananal et al., 2007; Furuya and Kinoshita, 2008; Bril and Goasdoué, 2009; Furuya et al., 2009, 2011; Kazennikov and Wiesendanger, 2009; Konczak et al., 2009; Verrel et al., 2013a, b; Schoonderwaldt and Altenmüller, 2014). This research was partly fueled by the necessity to study professional pathologies of musicians and to develop relevant therapeutic methods (Bragge et al., 2006; Kelleher et al., 2013).

In these studies of music performance, the registration methodology includes high-precision optic systems with the sensors fixed on the segments of the arm. In the case of string instruments the movements of the bow and of the corpus of the instrument are also registered, sometimes completed by pressure sensors and accelerometers fixed to the bow. This permits exhaustive analysis of, firstly, the movements of the bow in relation to the corpus of the instrument (violin, viola, or cello), and, secondly, movements in the arm joints.

Participants in these studies were music players of different levels of skill, from beginners to amateurs to professionals. Researchers examine the adaptation of joint coordination to various conditions of performance, including tempo (Winold et al., 1994; Rasamimananal et al., 2007; Furuya et al., 2011; Schoonderwaldt and Altenmüller, 2014), sound power (Furuya and Kinoshita, 2008; Furuya et al., 2009; Schoonderwaldt and Altenmüller, 2014), type of instrument (Turner-Stoker and Reid, 1999; Bril and Goasdoué, 2009), and character of partition (Winold et al., 1994). The abovementioned works demonstrate efficiency of “bottom-top” approach for the analysis of the differences in the level of skills, in both kinematic and dynamic aspects of the organization of movement. Kinematic differences between the skilled experts and the learners can be seen, in particular, in relatively smaller amplitudes of the shoulder movements (Konczak et al., 2009; Verrel et al., 2013b) and in proximal-to-distal gradients in the timing and joint amplitudes along the kinematic chain (Furuya and Kinoshita, 2008; Verrel et al., 2013a). By contrast, dynamic differences can be traced in the optimal usage of reaction forces in the joints like minimizing muscular forces of keystrokes (Furuya and Kinoshita, 2008; Furuya et al., 2009).

Results of complex coordination (including movements of working points of the arm important for executing musical task) are analyzed (Kazennikov and Wiesendanger, 2009; Schoonderwaldt and Altenmüller, 2014). Although the organization of the arm DoFs is not included in the scope of these researches, they are model examples of discovering regularities in movements of the arm’s working points. This might provide a basis for going deeper into studies in motor control.

In their paper (Schoonderwaldt and Altenmüller, 2014), the authors studied repeating monotonous movements of the bow (clockwise, anti-clockwise and figure-of-eight) performed in various tempos and with varied sound power by highly professional musicians, music students and advanced amateurs. The coordination was assessed by the relationship of bow velocity, which mainly controls the amplitude of the string vibration, and the inclination of the bow relative to the violin. Movements were recorded using a passive optical motion-capture system. The violin and the bow were equipped with 5 markers each. In addition, the bow was equipped with a sensor for measurement of bow force and 3D accelerometer. All participants adapted the coordination to tempo and level of sound (forte or piano) in an individual way. The results did not indicate a significant distinction between participants of different level of expertise, or regarding the strategy of coordination, or the ability to adapt to increasingly difficult performance situations. At the same time the results did indicate a significant difference between groups with respect to stability of performance: the student group tended to be most stable, followed closely by the professional group, and at a larger distance by the amateur group.

Bimanual coordination in violin playing was studied in the work (Kazennikov and Wiesendanger, 2009): the authors analyzed synchronization between the movements of the left hand fingers (fingering) and the right hand movement performance (bowing). Bimanual coordination was assessed on the basis of the degree of synchronization in relocation of fingers along the fingerboard and by changing from one string to another in performing tone production. Registration was made by four infrared sensors fixed at the working points of the arm – on the left-hand finger nails (index, middle, ring, and little fingers) – and by three sensors on the bow. It was shown that fingering and bowing are executed in parallel, rather than serially. No particular differences in the synchronizing of these movements between professionals and amateurs were detected.

Both studies mentioned above have not revealed reliable differences between various levels of professional training, not even between professionals and amateurs. It can be partly explained by the fact that all experimental subjects mastered the violin well, and the research method (analysis of working points movement) could not detect differences in performance. If difference in skills is more substantial, for instance, between novices and professionals, the analysis of working point movement shows substantial distinctions (Verrel et al., 2013a).

The only way to provide the right movement of the bow along the corpus of a violin or a cello is to organize DoFs of the arm adequately to the movement task. Even if the analysis of such organization is limited to two or three DoFs, it can show the difference in the level of skill. Thus, joint coordination during violin playing (examined in Konczak et al., 2009) reveals an expertise-related difference: the higher the violinist’s level, the smaller the contribution of shoulder flexion-extension in the bowing. In the study, three DoFs of the arm were analyzed: flexion-extension in the elbow and in the shoulder and abduction-adduction in the shoulder. However, the authors stressed that the wrist DoFs should also be taken in consideration.

The same result was obtained in the study of cello playing by experts and novices: novices showed a larger amount of shoulder variance, explained by the first principal component, compared with advanced players Verrel et al. (2013b). The outcome was confirmed by Bril and Goasdoué (2009): at a high level of skill the amplitudes of movements in the shoulder joint are substantially smaller than the amplitudes in the elbow joint. In the study comparing performances of the contemporary and the baroque violin, it was found that the differences are limited to the elbow joint and that the shoulder joint does not take part in the process of adaptation to the positioning the violin in relation to the persons’ body trunk and to the stiffness of cords and the bow.

The study of bow strokes performed at different frequencies (Rasamimananal et al., 2007) focused on coordination between the wrist and elbow joints. Two subjects performed bow strokes with altering tempo (accelerando–ritardando), one on violin, the other on viola. In this case, coordination between the wrist and elbow joints substantially differed with the pattern of in-phase and anti-phase periods. It remains unclear whether the difference in the patterns was due to the instrument used or to the individual character of the performer.

A more profound study of movement differences at various levels of skills can be achieved by analyzing a larger number of DoFs. Verrel and colleagues (Verrel et al., 2013a) investigated coordination of the right arm in a task requiring a sudden yet precisely controlled bow reversal during continuous tone production on a cello by experts and novices. Reflective markers attached directly on the trunk and on the right arm (acromion, lateral epicondyle of the elbow, forearm and hand) were used for movement recordings. Two additional markers were attached on the cello and the bow. The experimental setup allowed for the calculation of movements in all DoFs which in principle could be involved in the movement, but, following the information of the cello teaching literature, only shoulder adduction/abduction, elbow flexion/extension, and wrist and finger flexion/extension were taken in analysis as the most relevant joint angles. Joint angles were analyzed in terms of velocity and acceleration profiles, as well as in relation to temporal coordination along the arm.

The analysis of these parameters showed that experts, in contrast to novices, used differentiated coordination patterns, with proximal-to-distal gradients in the timing and amplitudes of acceleration peaks along the kinematic chain. The authors believe that, for a deeper understanding of the organization of the movement adequate to the task, one needs to analyze, besides the DoFs of the arm with the bow, the movement of the body (important both for the bow movements and for maintaining the balance). It is worth noticing that these studies (Verrel et al., 2013a, b) include the highest numbers of DoFs compared with other studies of arm movements in playing string instruments (Table 1). It is a good prospect for further research that the authors of these works consider increasing the number of DoFs under study.

**TABLE 1:** Motor studies of musical performance.

**Study**	**Type of movement**	**Arm joints**	**Number of DoFs**	**Movement goal**	**Level of expertise**	**Adaptation to constraints**	**Coordination (synergy)**
Schoonderwaldt and Altenmüller, 2014	Bowing, violin	–	–	Repeating monotonous movements in different tempo	Professionals, Students, amateurs	Tempo, level of sound	–
Kazennikov and Wiesendanger, 2009	Bowing, violin	–	–	Octave production	Professionals, amateurs	String changes, position changes	–
Rasamimananal et al., 2007	Bowing, violin, viola	Elbow, wrist	2	Détaché production in different tempo	Advances level students	Tempo	Wrist-elbow coordination (in-phase, anti-phase)
Bril and Goasdoué, 2009	Bowing, modern and baroque violin	Shoulder, elbow	2	Sound production, Gavotte in Rondo by J.S. Bach	Baroc violin players, modern violin players, both instruments players	Type of violin: violin modern vs violin baroque	–
Konczak et al., 2009	Bowing, violin	Shoulder, elbow	3	Sound production, “Twinkle, Twinkle, Little Star”	Children-learner, beginning-to-advanced level adult players, adult concert violinists	Sound production, staying on a fixed note	–
Winold et al., 1994	Bowing, cello	Elbow, wrist	2	Sound production, Sonata in E minor by J. Brams and Arpeggione Sonata, by F. Schubert	Highly skilled cellists	Tempo	Wrist-elbow coordination
Turner-Stoker and Reid, 1999	Bowing, violin, cello	Shoulder, elbow, wrist	3	Moderately slow legato bowing	Players across the range from student to mature professional	Type of instrument: cello, violin	–
Verrel et al., 2013a	Bowing. cello	Shoulder, elbow, wrist, finger	4	Continuous tone production	Professionals and novices	–	Proximal-to-distal gradient in professionals, with lower acceleration in more proximal joints
Verrel et al., 2013b	Bowing, cello	Shoulder, elbow, wrist, finger	4	Repeated bow movement at a prescribed tempo	Professionals and novices	–	Distal joints are temporally coupled in relation to the entire arm movement in professionals, not in novices
Furuya and Kinoshita, 2008	Keystroke, piano	Shoulder, elbow, wrist, MP joint	4	Octave production	Professionals and novices	Level of sound	Proximal-to-distal sequence in professionals
Furuya et al., 2009	Keystroke, piano	Shoulder, elbow, wrist, MP joint	4	Octave production	Professionals and novices	Level of sound	Expertise-dependent nature of gravity-muscular force interaction
Furuya et al., 2011	Keystroke, piano	Elbow pronation-supination, thumb rotation, fingers MCP flexion-extension	6	Tremolo with the thumb and little finger	Professionals and amateurs	Tempo	A smaller flexion velocity at the thumb and little finger and greater elbow pronation and supination velocity in professional comparing with amateurs.

Proximal-to-distal temporal patterns have also been described for piano keystrokes (Furuya and Kinoshita, 2008), during which end point velocity needs to be precisely controlled but not necessarily maximized. By contrast with other works, Furuya and colleagues do not limit their research to kinematics but include the dynamics of a multi-joint movement in their study. Inverse dynamic analysis (Furuya et al., 2009) completed by an analysis of electrical muscle activity (Furuya and Kinoshita, 2008) indicated that experts compared to novices generated greater muscle torques at proximal joints, exploiting resulting interaction torques to reduce distal muscle torques. The authors suggest that the expert musicians relied heavily on a gravity-dependent drop of the arm while keeping the contribution of the muscular force and work to a minimum (this relates to the shoulder joint, and not to the wrist). We can conclude, therefore, that the results correspond well with those achieved in Bernsteinian research (Bernstein and Popova, 1930) and that it is possible to take this historical work further.

Similar idea of simplified joint control using passive interaction of joint torques and of gravitational forces is developed in the study (Dounskaia and Wang, 2014). All seven arm DoFs during performing a drawing task were considered which allowed to show that the redundant DoFs enlarge the range of passive control application.

Like the pioneers of music movement studies (Demenÿ, 1905; Bernstein and Popova, 1930), contemporary researchers primarily use simple monotonous movements like staccato (Rasamimananal et al., 2007), continuous tone production (Kazennikov and Wiesendanger, 2009; Verrel et al., 2013a), repeated bow movement (Verrel et al., 2013b; Schoonderwaldt and Altenmüller, 2014), and playing octaves (Furuya and Kinoshita, 2008), and tremolo (Furuya et al., 2011). Performing music fragments, which implies a more meaningful movement task, could be the next step in studying movement coordination. Thus (Konczak et al., 2009), experimental subjects were asked to play the well-known eighteenth-century English nursery rhyme “Twinkle, Twinkle, Little Star.” In other research (Bril and Goasdoué, 2009), the subjects were to perform a Gavotte and Rondo by J.S. Bach, or (Winold et al., 1994) they played the Sonata in E minor, First Movement by J. Brahms and the Arpeggione Sonata, First Movement by F. Schubert. Winold and colleagues studied coordination between movements in the wrist joint and in the elbow joint (Winold et al., 1994). It was found that in different professional performers coordination is more stable in playing meaningful music works rather than while doing monotonous strikes in the same tempo. The authors of the study suggest that choice of coordination depends on the meaning of musical work, and the stability of coordination is due to the fact that professional performers, participants in the experiment, interpret music in a similar way. Compared with meaningful music fragments, monotonous strikes impose fewer limitations on the movements of the arm joints.

It can be suggested that the movements in the wrist and elbow joints rather roughly describe the cellist’s arm movements, and that the stable coordination can only account for the high level of skill. We can also suggest that a study of all DoFs would reveal nuances which distinguish the performer’s individual style.

Although in many works mentioned above the number of sensors and their position on the arm make it possible to analyze all seven DoFs, the number of DoFs in fact analyzed is everywhere reduced (Table 1). Playing string instruments puts mechanical limitations to the DoFs of the arm which are actually used. For instance, the authors (Konczak et al., 2009) believe that these limitations exclude rotation around the longitudinal axes of the shoulder and the forearm. Besides this rotation, other researchers (Verrel et al., 2013a) exclude also flexion in the shoulder joint and adduction of the hand (they refer to the information in cello manuals). Another point of view is that a closer definition of movement, for example to include pronation and supination at the elbow, ulnar and radial deviation at the wrists, or fine hand and finger movements, is likely to prove necessary to identify subtle differences in technique (Turner-Stoker and Reid, 1999). The latter point of view appears the most adequate. Playing violin, cello and piano naturally implies limited classes of arm movements. Inside these classes, some DoFs can be limited in amplitude. However, to leave them out of analysis could be an unjustified simplification.

Reducing the number of DoFs can limit the variability of movement, which in itself is am important characteristic of the level of skill. An obvious source for the variability is motor redundancy which is functionally necessary for a flexible adaptive behavior. To describe an adequate approach to the organization of motor control, Latash and Gelfand suggested speaking of “abundance” instead of “redundancy” (Gelfand and Latash, 1998; Latash, 2012). In accordance to it, all DoFs always participate in all the tasks, assuring both stability and flexibility of the performance. No DoFs are ever eliminated or frozen but dissociated in task important combinations – essential or functional DoFs – and combinations less important for a task – non-essential or non-functional DoFs. CNS resists more strongly those spontaneously occurring perturbations that change the value of the essential task variables, while perturbations that leave the same value invariant are resisted less strongly. In another words, CNS allows the DoFs to show high variability as long as it does not affect the desired value of the task important variables.

Bernstein saw “abundance” in movement’s *variability*, or *versatility*, not just in the very fact of multiple DoFs. “Even in standard acts like walking acquired in early childhood, when one passes from eye-viewing to using precise registration devices, it becomes clear that none of the steps is identical to any other. It is the case even when one walks on a straight path, not speaking of an uneven one” (Bernstein, 1961/1966, p. 277). Thanks to motor abundance, the organism learning a skill has the opportunity to try on a particular movement “to satiety,” repeating the same movement again and again without the repetition being identical. As a result, more and more DoFs become involved in the movement which the organism experiences as relaxation or “liberation.” This sensation also signals that the skill has been formed. The *feeling of freedom*, which arises when the organism can play with movements, is due to the abundance of *degrees of freedom*. This is more than just a pun on the word *freedom*: this is how the real process of learning happens, the process that divides complex life forms from simple ones.

## Highly Coordinated Stone-Knapping

Variability underlies both the process of learning skills and improving and perfecting them. Organization of hammer stroke in stone knappers of different level of skill (Biryukova and Bril, 2008; Biryukova et al., 2015) is described below in order to illustrate the changes in variability structure in the process of skill perfection.

Special strokes important for the final oval shape of the stone were selected from multiple hammer strokes performing during stone processing and then analyzed. The knappers were divided into four groups: high-level experts, low-level experts, high-level learners, and low-level learners. Arm movements during a hammer stroke were recorded with four electromagnetic sensors (Spatial Tracking System, Polhemus) attached the hand, the forearm, the arm and on the acromion of the knappers. Seven joint angles corresponding to arm DoFs were calculated (Biryukova et al., 2000): abduction–adduction (Ab-Ads), flexion–extension (F-Es), and rotation (Rots) around the longitudinal axis of the shoulder in the shoulder joint, flexion–extension (F-Ee) and pronation–supination (P-Se) in the elbow joint, and flexion–extension (F-Ew) and abduction–adduction (Ab-Adw) in the wrist joint. To calculate the movements of the hammerhead STS the sensor on the hammer handle and the accelerometer on the hammerhead were used.

Variability of movements in the arm joints was examined using principal component analysis. Joint angle loadings on the first principal component were considered a kinematic content of movement. Multidimensional, non-parametric scaling techniques of representation of mutual positions of the points in the seven-dimensional space of arm DoFs on the plane were used to assess variability of the contents of the stroke kinematics. The method (1) provides minimal distortion (in the sense of minimal sum of squares) of the distances between the points in the space of arm DoFs, and (2) it reveals the structure of the initial set of points in the form of groups or clusters (Kruskal, 1977).

**FIGURE 4:**
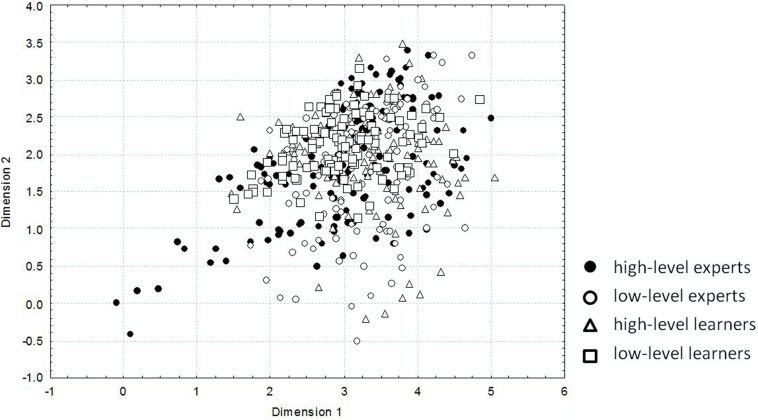
The projections on a plane of 7-dimensional space of joint angle loadings on hammerhead movement. The conditional dimensionless values – results of non-parametric multidimensional scaling – are set on the axes. Each point represents the projection of kinematic content of the stroke important for the final oval shape of a stone. Points corresponding to the stroke of high-level experts are shown by black circles, of low-level experts by white circles, of high-level learners by white triangles and of low-level learners by white squares. (Reproduced from Biryukova et al., 2015 with a permission from Human Kinetics Publishers).

The projections of kinematic content of the strokes are shown in Figure 4. A decreasing of variability with a decreasing of mastering of skill takes place: high-level experts demonstrate the largest variability (black circles), low-level experts a little smaller variability (white circles), high-level learners show still smaller variability (white triangles) and low-level learners the smallest one (white squares). In other words, the highest level of expertise is characterized by the use of a greater number of DoFs and by flexible joint configurations.

There are six functional variables of the knapping task - three coordinates and three Euler angles of the hammerhead position and orientation - that are enacted by seven independent arm DoFs. The redundancy equal to the difference between the number of DoFs and the number of functional variables is equal to 1. Despite a rather low redundancy, the variety of motor solutions in knappers of different levels of skill is very large (Figure 4). It is important to note that the *a priori* reduction of DoFs to a single instance results in a unique motor solution of the task, which makes the analysis of variability senseless.

Two components were distinguished in each movement of the joint: the functional, affecting the motion of the hammerhead, and the non-functional, with no influence on the movement of the hammerhead. The components were obtained using the *uncontrolled manifold theory*, *UCM* (Scholz and Schöner, 1999), an effective tool for dissociation of functional and non-functional components of goal directed movement. UCM was successfully applied to an analysis of 7-DoFs of the arm during pistol shooting (Scholz et al., 2000), throwing task (Yang and Scholz, 2005), end-state control in reaching (Solnik et al., 2013).

**FIGURE 5:**
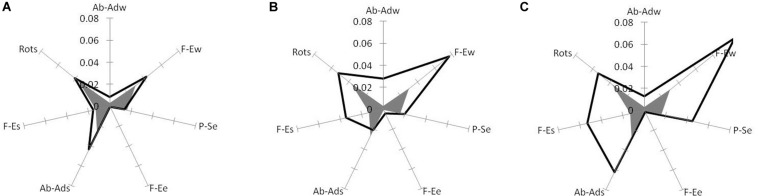
Non-functional joint variations in high-level experts (gray heptagon) compared with **(A)** low-level experts, **(B)** high-level learners and **(C)** low-level learners. Tops of the heptagons correspond to the contributions in non-functional component (squared and averaged over trials) of arm DoFs: abduction–adduction (Ab-Ads), flexion–extension (F-Es), and rotation (Rots) around the longitudinal axis of the shoulder in the shoulder joint, flexion–extension (F-Ee) and pronation–supination (P-Se) in the elbow joint, and flexion–extension (F-Ew) and abduction–adduction (Ab-Adw) in the wrist joint. (Reproduced from Biryukova et al., 2015 with a permission from Human Kinetics Publishers).

It became clear that, in spite of the wide spectrum of motor solutions that the experts used, movements of the joints were organized in a maximally “useful” way. In other words, despite the variety of trajectories, all joint movements essentially contributed to the motion of the hammerhead. The non-functional component is the smallest in high-level experts, and increases with a decrease of mastery of skill (Figure 5).

The contributions of the seven DoFs of the arm in the non-functional component represent the portions of joint angle variations that do not contribute to the hammerhead position and orientation (Figure 5). The non-functional joint variations as represented by the area within the heptagons of Figure 5 were the smallest in high-level experts (gray heptagons) and increased with decreasing level of motor skill. We consider the areas between heptagons as the representation of a kind of *potential for further learning of motor skill*. One can hypothesize that this area will diminish during learning that manifests more functionally justified use of joint angles. This representation shows that for both high-level (Figure 5B) and low-level (Figure 5C) learners this potential is quite high (Biryukova et al., 2015; see also Wagner et al., 2012; Hiley et al., 2013).

Bernstein claimed that variability of motor acts continuously transformed something that could be simple, mechanical repetition, into a play of variations, almost like performing and interpreting music. Variability therefore serves more than the purpose of adaptation: motor versatility contributes *modes and styles* of movement: “A number of cyclic movements, like walk, running, filing, flying movements of a bird’s or an insects’ wing can be well approximated by not so complex kinematic equations, the fact that speaks for their wholeness or interconnectedness, from the beginning to the end. The most emphatic might be the well-known fact that that movement skills like hammer stroke, running, all kinds of athletic exercises cannot be performed “as one likes”; they are molded in a small number of discreet forms without passages between them, which are called “styles” in sport and “skills,” or “operational modes,” in working movement” (Bernstein, 1961/1966, p. 278). Thus, variability lies at the foundation of both learning and improving skills and of creating styles in sport and, possibly, art. Finally, one can do justice to the refinements of motor control only by analyzing *all DoFs of a highly coordinated movement*. Further, we try to show that such analysis can also be productive in cases of pathology and loss of coordination, that is, in the case of the opposite of highly coordinated acts.

## Pathological Movements in Post-Stroke Patients

Bernstein’s earliest publication on motor pathology was his paper, “Clinical paths of contemporary biomechanics,” and it remains relevant almost a century later. He categorized kinematics of movement as a “large section of *morphology* in the wide meaning of the term,” something that describes the state of motor function in general (Bernstein, 1929). Normally movement is an integral process of coordination formed ontogenetically and determined by both anatomical (and biomechanical) individual features and the specificities of motor control, including the reaction of the central nervous system to external or peripheral forces. Kinematics of movement, he suggested, was the natural solution of the differential equations of movement dynamics that have been naturally integrated, and that was dependent on the alteration of muscular efforts.

Biomechanical parameters of movement for estimating degrees of motor impairment after central damage are widely used today (Alt Murphy and Häger, 2015). However, one cannot relate these researches to “morphology in the wide meaning of the term” for several reasons. Experiments are mostly limited to two or three DoFs, usually the coordination between flexions–extensions in the elbow and the shoulder joints (e.g., Levin, 1996; Beer et al., 2004; Micera et al., 2005). Rarely, abduction–adduction in the shoulder is added (e.g., Michaelsen et al., 2004). Yet in the majority of experiments coordination is judged indirectly, by pathologies of the end-point control (for instance, in the movement of the hand toward the goal) (e.g., Rodrigues et al., 2017).

At the first glance, this state of affairs may appear paradoxical: after a century, systems of registering movements have improved enormously and techniques of data analysis have been automated. Nevertheless, the analysis of multi-joint movement has not become easier: high-precision optic systems have turned out to be cumbersome and expensive, while the data obtained require complicated processing. As a result, laboratories – even those that can afford the equipment – use sensors just for the centers of joints, and often a single sensor is installed at the working point of the hand. This automatically excludes registering rotations about the longitudinal axes of the links.

The second reason why the number of analyzed DoFs remains limited is the kind of *movement tests* used for assessing motor function. These tests are mostly based on *reaching and grasping* movements, the kinematic structure of which does not include all DoFs of the arm. Pronation–supination of the forearm and rotation of the shoulder around the longitudinal axis, DoFs not only functionally important but also symptomatic of the recovery process, remain out of the experimenter’s view. In a way, researchers become hostages of the accepted tests of reaching and grasping, and they often do not reach the goal of coordination analysis and do not fully grasp the essence of coordination. Not accidentally, the review article about this mentioned above (Alt Murphy and Häger, 2015) is titled, “Kinematic analysis of the upper extremity after stroke – how far have we reached and what have we grasped?”

What offers an alternative to the standard movement tests? We propose a system of tests consisting of isolated movements following each DoF of the arm, that is, a *kinematic portrait* of the patient (Kondur et al., 2016). The tests consist of performing isolated movements for each DoF. They were used for assessing the efficiency of a promising rehabilitation procedure that uses an exoskeleton controlled by the brain-computer interface, based on somatosensory feedback (Frolov et al., 2017). An isolated movement requires a complex synergy in order to control all the DoFs except for the one included in the instruction to the test. After a stroke, an isolated movement is the first to suffer (Brunnstrom, 1970). The degree to which it suffers can have a diagnostic value in estimating the chances for recovery of multi-joint movement coordination. The examination of all DoFs of the arm helps in detecting small changes in DoFs, especially in cases of severe paresis. This is important for assessing the efficiency of therapeutic procedures (including the use of the brain-computer interface controlling exoskeleton) and selecting particular methods of rehabilitation (Biryukova et al., 2016; Kondur et al., 2016).

**FIGURE 6:**
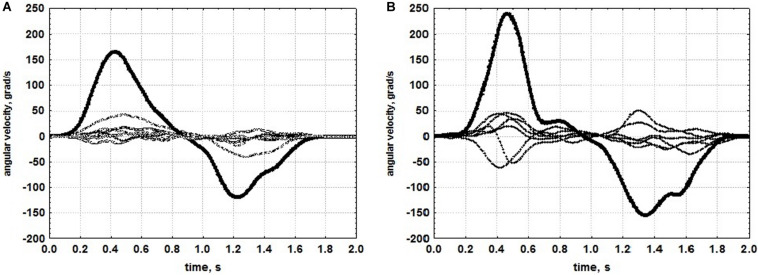
Time courses of velocity of isolated pronation-supination in a patient with moderate paresis. **(A)** Before rehabilitation using an exoskeleton of the hand controlled by brain-computer interface. **(B)** After rehabilitation. Bold lines denote angular velocity of pronation-supination, thin lines denote angular velocities in other arm DoFs. (Reproduced from Kondur et al., 2020 with a permission from Akademkniga Publishers).

**FIGURE 7:**
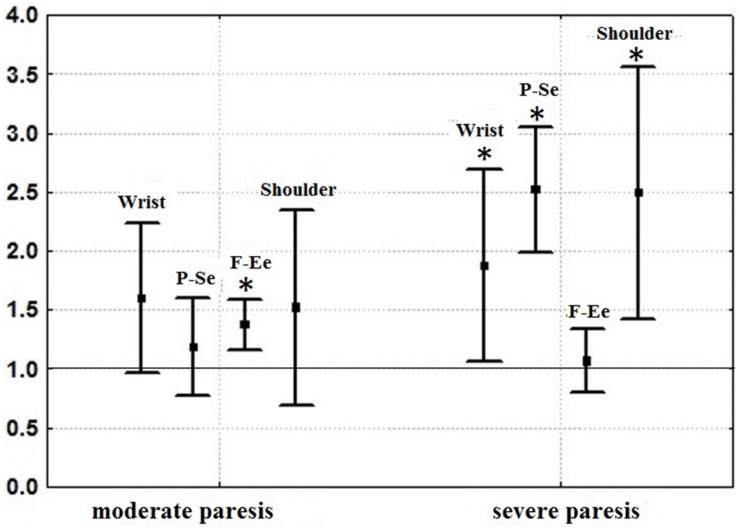
Mean standard deviations of angular velocities of isolated movements in the wrist joint (the sum of velocities of abduction-adduction and flexion-extension), of isolated flexion-extension in the elbow (F-Ee), of isolated pronation-supination of the forearm (P-Se) and of isolated movements in the shoulder joint (the sum of velocities of abduction-adduction, flexion-extension and rotation about longitudinal axis of the arm). The values of angular velocities before rehabilitation using an exoskeleton of the hand controlled by brain-computer interface are taken for one (thin horizontal line). Changes of angular velocities after rehabilitation relative to the values before rehabilitation are shown for the cases of moderate (5 patients) and severe (6 patients) paresis (results of ANOVA analysis). The asterisks denote a statistically significant increase of angular velocities. (Reproduced from Kondur et al., 2020 with a permission from Akademkniga Publishers).

An example of one motor test from the kinematic portrait, an isolated pronation-supination, the DoF which suffers the most during spastic hemiparesis (Kadykov et al., 2014), is given in Figure 6 (from Kondur et al., 2020). The patient with moderate paresis, who has undergone rehabilitation using a hand exoskeleton controlled by the brain-computer interface, demonstrated an increase in angular velocity of pronation-supination (Figure 6). Angular velocities of isolated movements corresponding to other DoFs also increased (Figure 7), which gives evidence for the efficiency of the rehabilitation procedure. Relative increases of angular velocities were found to be greater in patients with severe paresis (six clinical cases analyzed) than in patients with moderate paresis (five clinical cases analyzed). In addition, the increase was statistically significant for more DoFs in patients with severe paresis compared to the patients with moderate paresis. It turned out that the most impaired pronation-supination showed the most effective recovery. Rehabilitation using the brain-computer interface based on somatosensory feedback was initially designed for patients with severe paresis, as it appeared the only possible active paradigm of rehabilitation for patients with significant motor deficit (Buch et al., 2008; Daly and Wolpaw, 2008). The data presented in Figures 6, 7 confirm the effectiveness of this kind of rehabilitation for patients with severe paresis.

The need of quantitative methodology of studying the neural control of multiple DoFs is discussed in connection with studies of pathological movement (Santello and Lang, 2015). Kinematic portrait testing each arm DoF can be considered as a kind of such methodology.

Despite of limited number of analyzed DoFs biomechanical analysis of movements in patients after central paresis should be considered as advanced method because it provides objective numerical assessment of motor function. Commonly accepted clinical methods of motor function assessment are the clinical scales which use the discrete number of points (as usual, 0,1,2) for an assessment of different motor tests (Fugl-Meyer et al., 1975; Gladstone et al., 2002). These assessments are naturally subjective and, which is more crucial, insufficiently sensitive to small changes in motor function. At the same time, the motor tests used in the widely used clinical scale Fugl-Meyer are well elaborated and representative for an assessment of the state of arm synergies (extensor, flexor, movements out of synergy etc.). Registration and biomechanical analysis of clinical motor tests could be, in our opinion, an effective tool of an assessment of motor function recovery after central paresis (Dzhalagoniya et al., 2018).

Bernstein’s program for studying movement pathology (Bernstein, 1929) included, besides the analysis of kinematics of movement, the examination of motor control exerted by the nervous system, following “bottom-up” approach. Bernstein described the nervous structures responsible for motor control as “biomechanical range” (*areal*, in Russian). The biomechanical areal is more than just one nervous center; it is a cluster of elements of the central nervous system, a substrate for core components of the nervous impulse that control the given movement. Bernstein gave an example of the biomechanical range in *pallidum tremor*. Frequency and amplitude of tremor differ from each other according to their cerebral origins: while frequency is entirely determined by the *pallidum* component of the biomechanical range, amplitude is linked to the innervations of voluntary movements coming outside the areal. Finding the biomechanical areal of a particular movement can be a painstakingly slow process.

In spite of considerable progress in research on movement as the indicator of nervous activity (Biryukova, 2011), after a hundred years we know only some biomechanical ranges for very simple rhythmical movements. For instance, analysis of the data of functional magneto-resonance tomography for flexion-extension in the elbow joint demonstrated (1) a correlation between the primary sensory cortex activity and the amplitude of flexion–extension, and (2) a correlation between the neuron net’s activity in the cerebellum and frontal lobes and the movement variability (Van Dokkum et al., 2017). Another example of “bottom-up” approach is the study of the representations of dexterous finger movements at the brain areas using a non-invasive transcranial magnetic stimulation (TMS). A comparison of hand movements elicited by TMS over the primary motor cortex between pianists, violinists and non-musicians identified distinct movement features associated with the trained movement repertoire (Gentner et al., 2010). To reconstruct accurately movement repertoire a small subset of TMS induced movements was sufficient. The conclusion is that the motor system may coordinate even the most dexterous movements by using a modular architecture involving cortical components (Gentner and Classen, 2006).

The reason why studying the nervous system by means of movement analysis should be so difficult is the incorrectness of the *inverse problems*: muscular forces are not unequivocally determined by the kinematics of movement, signals from the nervous system to the muscles cannot be unequivocally calculated on the basis of muscular forces and electromyographic activity. Bernstein’s approach to the inverse problem was the following: “*studies of localization should start not with answering the question*, ***where*** *something is located, but* ***what*** *is located, which exactly function [of the body], and* ***how*** *the function is reflected.*… *It should start with a correct definition of* ***categories*** *that can be located in brain centers*” (Bernstein, 1935/1966, pp. 54, 57, the emphasis in the original). Defining *whats* of multi-joint movements is the next step in examining brain functions by means of movement analysis.

The loss of coordination due to central nervous pathology means a lesser complexity of movement. A post-stroke patient can willfully use only a few of the possible DoFs. The objective of rehabilitation is therefore to restore both control over movement and movement complexity to the patient. In actual fact, this means helping the patient to learn anew how to coordinate movement. Thanks to neuroplasticity, in recovery and rehabilitation intact areas of the brain learn to perform motor control functions that earlier were not theirs. If the motor function is seriously damaged and the patient is unable to perform the movement, rehabilitation can employ the patient’s *kinesthetic imagination* (recollecting and imagining particular kinesthetic sensations when the desired movement is performed). Kinesthetic imagination is close to *anticipation*, the process of envisaging the outcome of a movement. Bernstein considered anticipation (which he termed the “model of desired future”) the main constructive element of movement planning and the starting point for organizing the entire movement (Bernstein, 1961, 1966). Methods of rehabilitation involving kinesthetic imagination should be therefore adequate to the rehabilitation goal (Ramos-Murguialday et al., 2013; Ang et al., 2014, 2015; Ono et al., 2014; Frolov et al., 2017). If rehabilitation is successful, the arm re-learns new coordinated movements in the joints; as in the initial acquisition of the skill, the movements “come by themselves,” naturally. New coordination may not be identical with the “pre-stroke” ones. The rehabilitation task can be considered solved if new coordination fulfil the patient’s functional needs (Latash and Anson, 1996).

As in the case of learning a movement skill, a new coordination comes as a whole in the recovery of movement. Bernstein emphasized two important points of recovery: involving in the process of all DoFs of the limb and strictly individual character of motor function recovering (Bernstein and Buravtseva, 1954; translated in Wagenaar and Meijer, 1998). As a consequence, to assess objectively the efficiency of rehabilitation therefore requires biomechanical analysis of all DoFs of the arm. An analysis that reduces DoFs is not adequate to the organizational principles of movement construction.

## Conclusion

Bernstein believed that in order to understand how the nervous system exercises motor control one should analyze multi-joint movements: “…motor acts are now interpreted as unconditional and direct outward reflections of the processes in the central nervous system… A more complex and flexible indicator, <motor acts> need more elaborate decoding procedures…*Yet this is a question of technique, and not of principle*, a question of the coming of a new Champolion who will interpret these hieroglyphs” (Bernstein, 2004, published in Feigenberg, 2004, p. 199, italics added – E.B., I.S.).

Yet his ambitious program has not been matched by later research. The main difficulty in examining the nervous system by means of movement analysis is, speaking in mathematical terms, *incorrectness of the inverse problems*. There is no unique solution of these problems; however, the number of possible solutions is limited by the construction of bone-muscular apparatus and the specificity of functions of brain structures. To explore further the heuristic potential of inverse problems, biomechanics and motor control should not be considered separate subject fields. We agree with Mark Latash that their separation is an “atavism” (Latash, 2016, p. 18).

Bernstein’s idea of exploring movements as an indicator of processes in the central nervous system could provide both a theoretical framework for further research and an instrument for assessing the efficiency of rehabilitation procedures. Bernstein studied nervous control with the “bottom-up” approach, and he used natural experiment in which a disease, or pathology, is considered an experiment designed by nature. In Bernstein’s experiments movement was not either reduced or simplified, as it has been in many other experiments performed both before and after him. He designed his experiments with intelligence and used thorough mathematical analysis; both helped in formulating his *theory of movement construction* and, later, *physiology of activity*. The program of studying the nervous system by analyzing complex movement is a frontier of neurosciences yet to be achieved.

## Author Contributions

Both authors: substantial contributions to the conception of the work; or the interpretation of data for the work; Drafting the work or revising it critically for important intellectual content; Final approval of the version to be published; Agreement to be accountable for all aspects of the work in ensuring that questions related to the accuracy or integrity of any part of the work are appropriately investigated and resolved.

## Conflict of Interest

The authors declare that the research was conducted in the absence of any commercial or financial relationships that could be construed as a potential conflict of interest.
